# New tumour entities in the 4th edition of the World Health Organization Classification of Head and Neck tumours: odontogenic and maxillofacial bone tumours

**DOI:** 10.1007/s00428-017-2182-3

**Published:** 2017-07-03

**Authors:** Paul M. Speight, Takashi Takata

**Affiliations:** 1 Unit of Oral and Maxillofacial Pathology, School of Clinical Dentistry, University of Sheffield, Claremont Cresent, S10 2TA UK; 2 Department of Oral & Maxillofacial Pathobiology, Institute of Biomedical & Health Sciences, Hiroshima University, 1-2-3 Kasumi, Minami-ku, Hiroshima, 734-8553 Japan

**Keywords:** Odontogenic tumours, Odontogenic cysts, Fibro-osseous lesions, WHO classification, Maxillofacial pathology

## Abstract

The latest (4th) edition of the World Health Organization Classification of Head and Neck tumours has recently been published with a number of significant changes across all tumour sites. In particular, there has been a major attempt to simplify classifications and to use defining criteria which can be used globally in all situations, avoiding wherever possible the use of complex molecular techniques which may not be affordable or widely available. This review summarises the changes in Chapter 8: Odontogenic and maxillofacial bone lesions. The most significant change is the re-introduction of the classification of the odontogenic cysts, restoring this books status as the only text which classifies and defines the full range of lesions of the odontogenic tissues. The consensus group considered carefully the terminology of lesions and were concerned to ensure that the names used properly reflected the best evidence regarding the true nature of specific entities. For this reason, this new edition restores the *odontogenic keratocyst* and *calcifying odontogenic cyst* to the classification of odontogenic cysts and rejects the previous terminology (keratocystic odontogenic tumour and calcifying cystic odontogenic tumour) which were intended to suggest that they are true neoplasms. New entities which have been introduced include the *sclerosing odontogenic carcinoma* and *primordial odontogenic tumour*. In addition, some previously poorly defined lesions have been removed, including the ameloblastic fibrodentinoma, ameloblastic fibro-odontoma, which are probably developing odontomas, and the odontoameloblastoma, which is not regarded as an entity. Finally, the terminology “cemento” has been restored to *cemento-ossifying fibroma* and *cemento-osseous dysplasias*, to properly reflect that they are of odontogenic origin and are found in the tooth-bearing areas of the jaws.

## Introduction and background

The principle of developing an international standard for the classification of tumours was agreed by the WHO in 1952, but the 1st editions of the International Histological Classification series were not published until after 1967. Number 5 in the series, published in 1971, was the first attempt at an internationally agreed standard classification of odontogenic tumours [1]. This 1st edition was titled “Histological typing of odontogenic tumours, jaw cysts and allied lesions”, and was deliberately inclusive, to ensure that all neoplasms and cysts of the odontogenic apparatus were classified in context, so that pathologists would appreciate and understand the commonly shared features of these lesions and be able to reach an informed diagnosis. The classification also included a number of bone lesions that have distinctive features when arising in the jaws and which must be distinguished from odontogenic neoplasms. The 2nd edition was published in 1992 and maintained this broad scope [2]. The 3rd edition was published in 2005 [3] and excluded the odontogenic cysts but did include bone-related or “tumour-like lesions”. This was an unusual decision, which was not explained. While the authors appeared to recognise that bone lesions are important in the differential diagnosis of lesions of the jaws, they clearly felt that clarity over the odontogenic cysts was not needed. They also ignored the fact that there was still ongoing debate regarding the true nature of a number of lesions, which sat at the “cyst-tumour interface”. Not including these lesions in the classification caused uncertainty regarding the correct terminology and management.

The new 4th edition has reinstated the odontogenic cysts and has restored this books status as the only complete classification of lesions of the odontogenic tissues [4]. The overall approach by the editors and the international consensus group was to simplify the classification and to clarify terminology so that the names of lesions properly reflected their nature and biological behaviour and were clearly understood internationally. Each section in the chapter was written by a group of experts selected from different regions of the world, to ensure a global perspective and to account for regional differences in approaches to terminology and diagnosis. The final version of the classification was then agreed by an international consensus group^1^ after vigorous and sometimes heated debate. The primary aim of the group was to ensure that any changes, insertions or deletions were supported by sound evidence. This brief review will highlight the key changes in the new edition.

## What is new in the 4th edition

### Odontogenic cysts

The most striking and welcome change is the return of the odontogenic cysts. This classification is simple and very similar to that used in the 2nd edition [2] (Table 1). The classification avoids including variants of lesions, which although well recognised, do not constitute separate entities and do not impact on management. Thus, they are discussed in the text, but not listed in the classification. For example, “residual”, “apical” and “lateral” cysts are not included as distinct subsets of radicular cyst, and “eruption cyst” is included only as a variant of dentigerous cyst. In this respect, it should be noted that the WHO classification is not intended to be a definitive textbook, but rather a simple guide to terminology and definitions. More complex classifications, which may consider the pathogenesis of lesions and fine details of the clinic-pathological features of variants, can be found in specialist textbooks [5].

**Table 1:** Classification of odontogenic cysts

Odontogenic cysts of inflammatory origin
Radicular cyst
Inflammatory collateral cysts
Odontogenic and non-odontogenic developmental cysts
Dentigerous cyst
Odontogenic keratocyst
Lateral periodontal and botyroid odontogenic cyst
Gingival cyst
Glandular odontogenic cyst
Calcifying odontogenic cyst
Orthokeratinised odontogenic cyst
Nasopalatine duct cyst

The key elements to note about the classification of the cysts are that it restores the *odontogenic keratocyst* as a cystic lesion and also classifies *calcifying odontogenic cyst* as a benign cyst (see below). The orthokeratinised odontogenic cyst is also recognised as an entity rather than being regarded as a variant of the odontogenic keratocyst.

#### Odontogenic keratocyst

Odontogenic keratocyst (OKC) has been reinstated as the preferred term for this simple keratinising cyst. There is a very large literature recording debate around the putative neoplastic nature of this lesion. For the most part, this has been centred on its so-called “aggressive” behaviour and the fact that a proportion of lesions are associated with a mutation or inactivation of the *PTCH1* gene, which was cited as the key factor supporting the re-designation of OKC as a neoplasm [6]. Although *PTCH* alterations are seen in up to 80% of OKCs [7, 8], they are not specific, since loss of heterozygosity (LOH) on the 9q22.3 region (where the *PTCH1* gene has been mapped) have been found in other developmental cysts [9], including dentigerous cyst [10]. However, this work needs confirmation, and sequencing data on these lesions has not yet been presented. It has also been reported that marsupialisation is an effective treatment for the odontogenic keratocyst and may be associated with reversion of the epithelium to normal, and with lower recurrence rates [11, 12]—features not normally associated with neoplasia. In considering all the available data, the WHO consensus group concluded that further research is needed, but at the present time, there was insufficient evidence to support a neoplastic origin of the odontogenic keratocyst. It was decided therefore that odontogenic keratocyst remains the most appropriate name for this lesion, and keratocystic odontogenic tumour (KCOT) was removed from the classification.

#### Calcifying odontogenic cyst

**Fig. 1:**
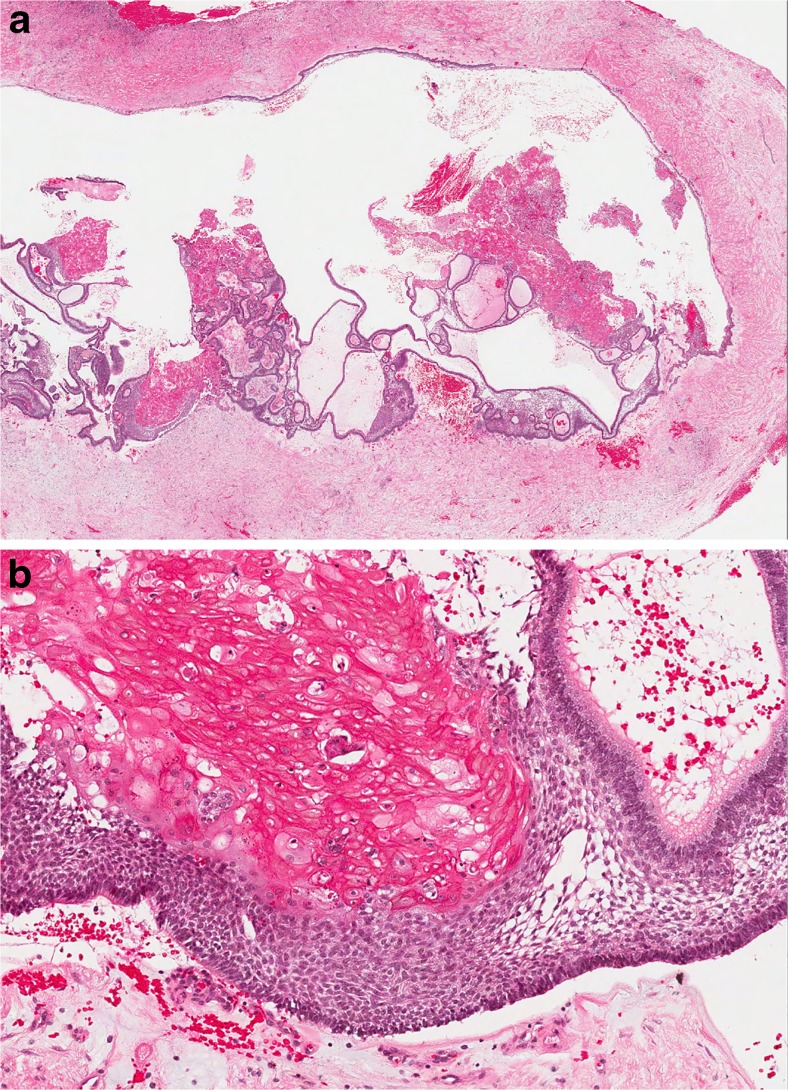
Calcifying odontogenic cyst. The lesion is unicystic but may show prominent luminal proliferations (**a**). The lining shows typical ameloblastomatous features, but ghost cells are the key diagnostic criterion for this lesion (**b**)

This lesion is a member of the “family” of ghost cell lesions [13]. In both the 1st and 2nd editions of the WHO classification [1, 2], it was listed under benign odontogenic tumours, but in 1971, it was clearly defined as a “non-neoplastic cystic lesion” [1]. In 1992, however, the authors seemed uncertain—they used an almost identical definition but removed “non-neoplastic” and defined it as a “cystic lesion”. In the text, however, they suggested that the cyst was non-neoplastic, but that a more solid variant was neoplastic and used the term “dentinogenic ghost cell tumour” [2]. In the 2005 edition, the calcifying odontogenic cyst (COC) was renamed as *Calcifying cystic odontogenic tumour* and was clearly defined as a “benign cystic neoplasm” [3]. The solid variant was included as a separate entity and termed *dentinogenic ghost cell tumour* (DGCT). However, the true nature of COC remains uncertain. In a detailed multicentre review of ghost cell lesions and their terminology, Ledesma-Montes et al. [13] showed that over 85% of ghost cell lesions are simple cysts either alone (65%) or associated with odontomas. Very few showed ameloblastomatous proliferations, and only 5% of lesions were solid and could be regarded as true neoplastic dentinogenic ghost cell tumours. These findings agreed with a previous study by Hong et al. [14], and both authors showed that simple cystic lesions rarely recur and have a completely benign course. Hong et al. described these lesions as simple cysts and only regarded solid lesions as true neoplasms. There seems, therefore, to be good evidence that simple cystic lesions should be regarded as developmental cysts, which arise alone or in association with other developmental lesions, especially odontomas [13–16]. In the new 4th edition of the WHO classification, the consensus group agreed to revert back to the original terminology and classify the cyst as calcifying odontogenic cyst and the neoplasm as dentinogenic ghost cell tumour. COC is therefore included under odontogenic cysts and DGCT under odontogenic tumours (Tables 1 and 2). COC is defined as a unicystic lesion, lined by ameloblastomatous epithelium containing focal accumulations of ghost cells. Luminal projections of ghost cells and ameloblastomatous epithelium may be seen, but mural proliferations are absent or minimal (Fig. 1) [17].

**Table 2:** Classification of odontogenic tumours

Malignant odontogenic tumours
Odontogenic carcinomas
Ameloblastic carcinoma
Primary intraosseous carcinoma NOS
Sclerosing odontogenic carcinoma^a^
Clear cell odontogenic carcinoma
Ghost cell odontogenic carcinoma
Odontogenic carcinosarcoma
Odontogenic sarcomas
Benign epithelial odontogenic tumours
Ameloblastoma
Ameloblastoma, unicystic type
Ameloblastoma, extraosseous/peripheral type
Metastasizing ameloblastoma
Squamous odontogenic tumour
Calcifying epithelial odontogenic tumour
Adenomatoid odontogenic tumour
Benign mixed epithelial and mesenchymal odontogenic tumours
Ameloblastic fibroma
Primordial odontogenic tumour^a^
Odontoma
Odontoma, compound type
Odontoma, complex type
Dentinogenic ghost cell tumour
Benign mesenchymal odontogenic tumours
Odontogenic fibroma
Odontogenic myxoma/myxofibroma
Cementoblastoma
Cemento-ossifying fibroma^a^

^a^New entities or terminology added since the 3rd (2005) edition

#### Orthokeratinised odontogenic cyst

**Fig. 2:**
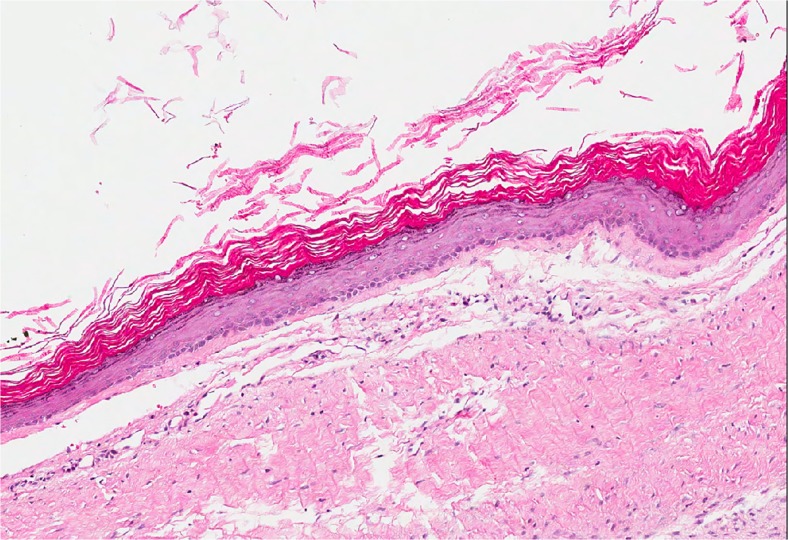
Orthokeratinised odontogenic cyst is lined by orthokeratinised epithelium with a prominent granular cell layer. Unlike OKC, the basal layer is not palisaded

The orthokeratinised odontogenic cyst was first described in 1981 as a variant of OKC [18] and was not included in previous editions of the WHO classification. The clinical presentation of orthokeratinised odontogenic cyst (OOC) is similar to OKC, often arising in the posterior mandibule, but radiographically, it most often appears as a well-circumscribed unilocular radiolucency [19, 20]. Similar to OKC, about half of lesions may be associated with an unerupted tooth giving an appearance similar to a dentigerous cyst. As the name suggests, histology shows an orthokeratinised stratified squamous epithelial lining with a prominent granular cell layer (Fig. 2). Although multiple OOC have been reported [21], there is no recorded case of lesions arising in association with the nevoid basal cell carcinoma syndrome. Also, unlike OKC, the lesions very rarely recur even after simple enucleation. OOC has now finally been classified as a distinct entity in the new WHO classification (Table 1).

### Odontogenic tumours

Both the 2nd and 3rd editions divided the odontogenic tumours into groups or subdivisions depending on the types of odontogenic tissues involved and on the degree of inductive change leading to hard tissue formation [2, 3]. However, the 3rd edition went further and divided some lesions into subtypes according to their putative biological origins—for example, primary type or secondary type, or according to histological variants. At times, the classification became too complex. For example, in the 2nd edition, 21 odontogenic tumours were listed, but this increased to 30 in the 3rd edition even though there was only one “new” entity (KCOT), and one lesion was excluded (carcinosarcoma). The new 4th edition has gone back to a more reductionist approach, and despite including three new lesions, it lists only 23 entities under odontogenic tumours [4]. The new classification divides the lesions into malignant and benign, and simplifies the subdivision of the benign tumours into those that are epithelial, mesenchymal or mixed, without attempting to describe the degree of inductive change. The classification of odontogenic tumours is shown in Table 2. There are a number of new entities, and some lesions that have been reinstated or renamed.

### New entities

#### Sclerosing odontogenic carcinoma

**Fig. 3:**
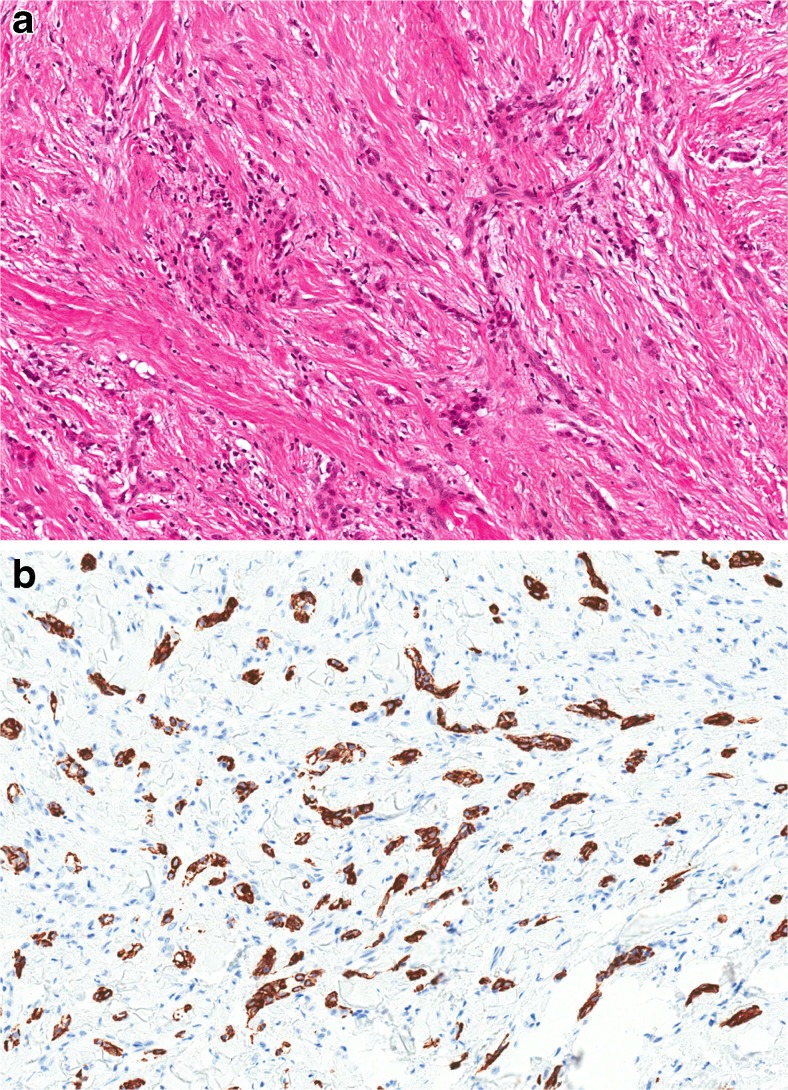
Sclerosing odontogenic carcinoma is characterised by strands and islands of epithelium infiltrating through a sclerotic fibrous stroma (**a**). The full extent of the epithelial component may only become apparent after immunohistochemical staining with a cytokeratin (**b**)

Sclerosing odontogenic carcinoma (SOC) was first fully described in 2008 [22], but further cases have subsequently been reported [23–26]. It is characterised by features of a low-grade malignancy with evidence of infiltration—it presents as a poorly defined radiolucency with evidence of bone destruction and tooth resorption. Histologically, it is composed of thin cords or strands of odontogenic epithelium permeating through a sclerosed fibrous stroma (Fig. 3). Occasional foci of clear cells may be seen. The lesion shows destruction of the cortical plates with invasion of adjacent connective tissues and muscle. Perineural infiltration is characteristic. Occasional lesions have been associated with calcifications resembling a fibro-osseous lesion [25]. No lesions have been reported to have metastasised.

The lesion has been controversial, and some have suggested that it should not be regarded as a new entity [27]. However, despite only a few case reports, the WHO consensus group felt that it was sufficiently distinct to deserve inclusion, but emphasised that further cases need to be reported so as to more accurately define its characteristics. The lesion may share features with odontogenic fibroma, primary intraosseous carcinoma, calcifying epithelial odontogenic tumour or clear cell odontogenic carcinoma; these should be excluded before a definitive diagnosis can be made. In this respect, we have recently found that, unlike clear cell odontogenic carcinoma (see below), SOC does not show EWSR1 rearrangements.

#### Primordial odontogenic tumour

**Fig. 4:**
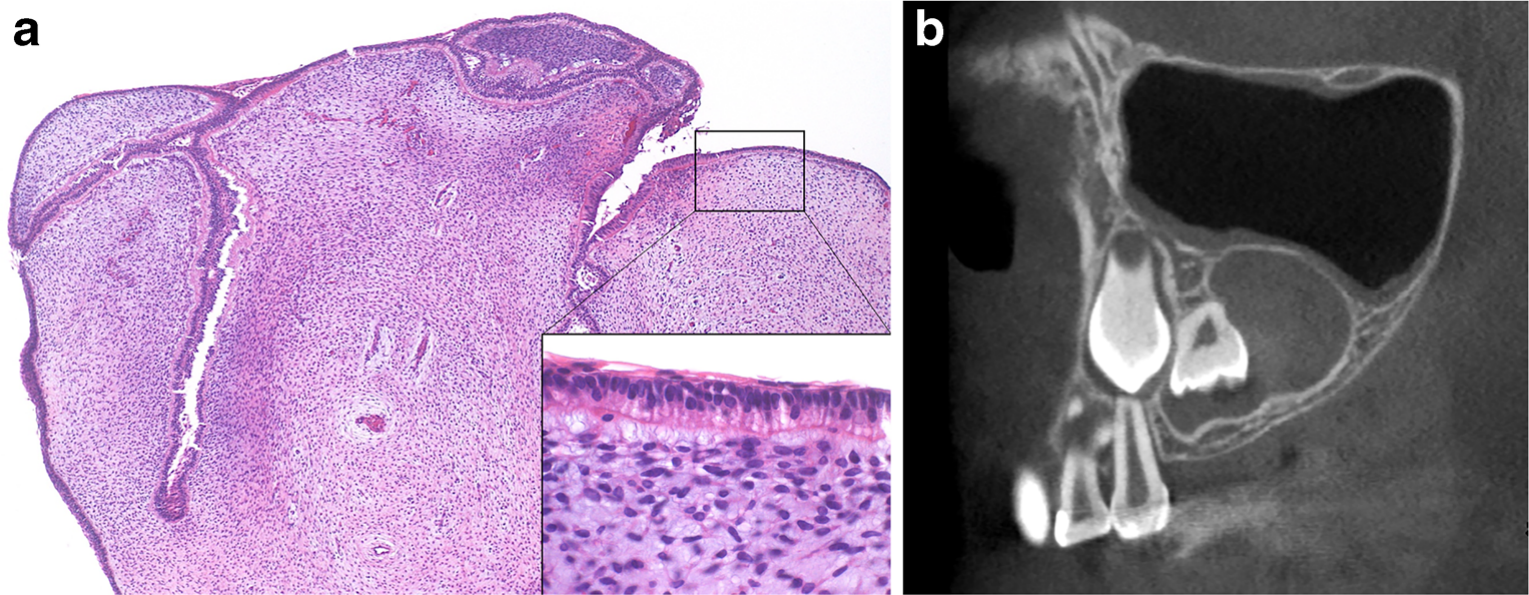
Primordial odontogenic tumour is composed of loosely cellular odontogenic mesenchyme surrounded by odontogenic epithelium (**a**). This resembles reduced enamel epithelium with columnar ameloblast-like cells (inset). This lesion arose in an 8-year-old girl and shows a well-demarcated radiolucency in a dentigerous relationship with an unerupted premolar tooth (**b**)

Primordial odontogenic tumour (POT) is a new entity with only seven reported cases. Six cases were first presented by Mosqueda-Taylor et al. in 2014 [28], with a further single case reported in 2016 [29]. It is a benign tumour composed of odontogenic mesenchyme with loosely arranged fusiform or stellate fibroblasts resembling dental papilla (Fig. 4a). The lesion is surrounded by a layer of cuboidal epithelial cells resembling the internal enamel epithelium. All lesions so far reported have presented as a well-demarcated radiolucency in a dentigerous relationship with an unerupted tooth (Fig. 4b)—most often a third molar. A recent study has more carefully analysed, four of the original cases using a panel of 23 antibodies [30]. The overall immunoprofile was consistent with a lesion of odontogenic origin and supports the view that it arises from the dental primordium—that is an abortive tooth germ that fails to develop into a dental organ.

#### Odontogenic carcinosarcoma

The odontogenic carcinosarcoma is not a new entity, since it appeared in the 2nd edition of the WHO book [2] but was not included in 2005. It has been included in the new edition, since, even though there are only a few single cases reports [31–34], there is no doubt that the lesion exists. Histologically, it is similar to *ameloblastic fibroma*, but both the epithelial and the connective tissue components, show clear cytological evidence of malignancy. Lesions have been reported to recur and to metastasise. The book does not venture into a discussion of the possible origins of these biphasic malignancies, but the author does emphasise that both components of the lesion must be frankly malignant and that the true carcinosarcoma should be distinguished from *ameloblastic carcinoma* with a malignant spindle cell component, which is probably associated with epithelial-mesenchymal transition and is properly referred to as sarcomatoid ameloblastic carcinoma [35].

### Changes in terminology

#### Ameloblastoma

The new edition has simplified the terminology around the ameloblastoma. In 2005, ameloblastomas were sub-divided into the *solid/multicystic type*, *extraosseous/peripheral type*, *desmoplastic type* and *unicystic type* [36]. This subclassification was regarded as too complex and lacking in behavioural or biological significance. The new classification has dropped the terminology “solid/multicystic”, since this pattern is well recognised as typical for conventional ameloblastoma, and use of “cystic” may cause confusion with the unicystic type. Desmoblastic ameloblastoma has also been dropped as a specific type and described as a histological variant of conventional ameloblastoma. Like other variants, including follicular, plexiform and acanthomatous, they are histologically distinctive and can be described, but as a diagnostic entity, there is no evidence of any differences in behaviour. Peripheral ameloblastoma does behave differently and is retained as a specific subtype.

**Fig. 5:**
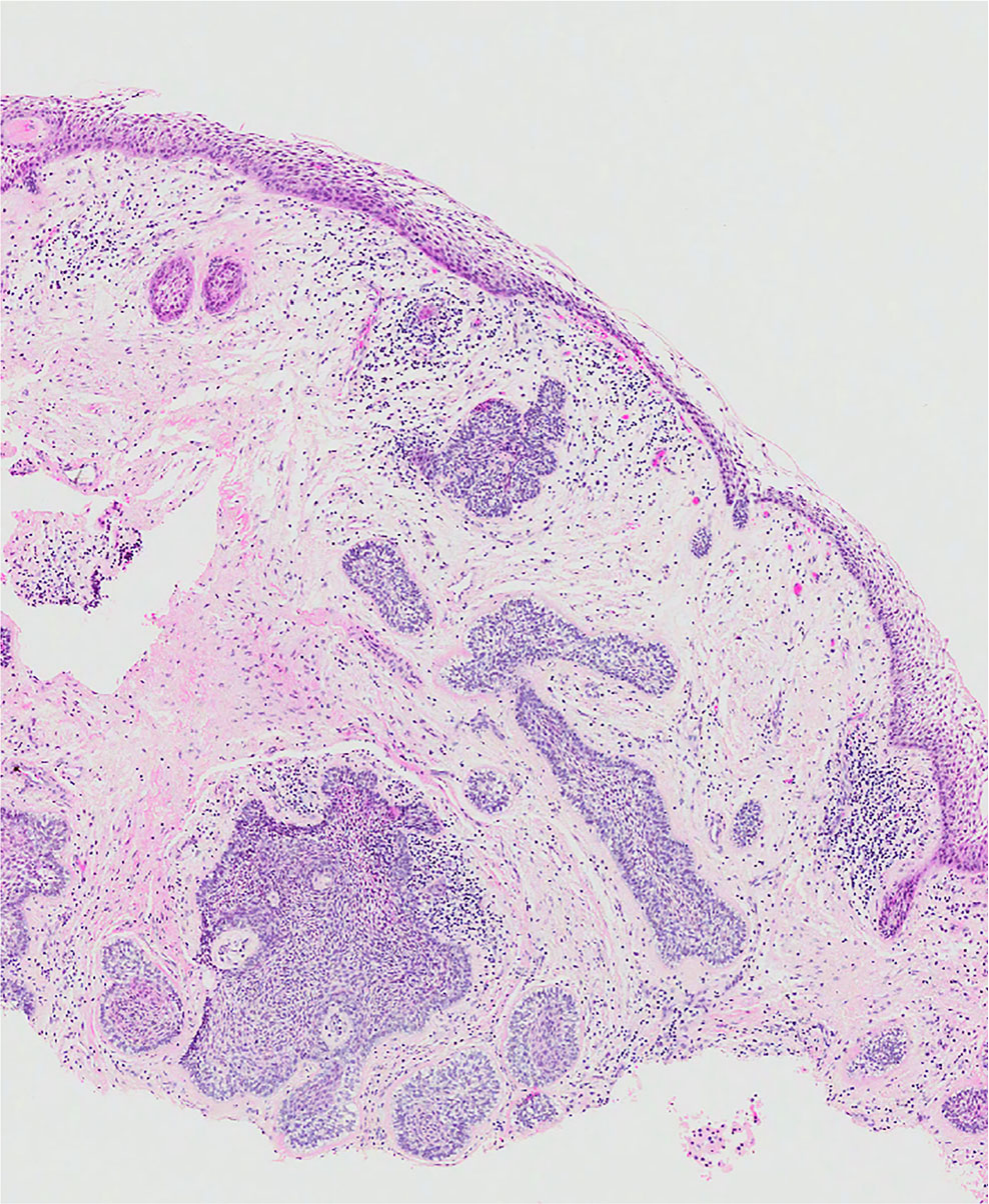
The wall of an enucleated cyst shows a lining of ameloblastomatous epithelium, but with prominent islands of follicular ameloblastoma in the wall. Should this be regarded as a “mural type” unicystic ameloblastoma or cystic change in a conventional follicular ameloblastoma?

Similarly, there is good evidence that unicystic ameloblastoma has distinct behavioural and clinicopathological characteristics, and it is retained as a subtype. The unicystic ameloblastoma is described as having three histological variants [37]. Two types are not controversial and are well recognised: the *luminal type* is a simple cyst lined by ameloblastomatous epithelium and the *intraluminal type* is similar but with luminal proliferations of (often plexiform) ameloblastomatous epithelium. These two types are considered to have a good prognosis and rarely recur even after simple enucleation. More controversially, the consensus group retained the *mural unicystic ameloblastoma* as one of the three types. This type shows proliferation of ameloblastomatous epithelium into the cyst wall (Fig. 5), and there is good evidence that it behaves in a similar manner to conventional ameloblastoma with similar recurrence rates [38]. However, despite some evidence of more aggressive behaviour, the group felt that further research was needed to clearly define the behaviour of this lesion, before a reclassification was justified.

The *odontoameloblastoma*, which was included as an entity in 2005, has been completely deleted. This is because there is no real evidence that it is a true mixed neoplastic ameloblastoma with odontogenic mesenchymal tissues. Rather these lesions represent a conventional ameloblastoma that happens to arise in association with an odontoma.

In this new edition, the *metastasizing ameloblastoma* has been moved from the section on ameloblastic carcinomas and has been included as a type of benign conventional ameloblastoma. It is defined as a histologically benign typical ameloblastoma which metastasises to distant sites. Both the primary lesion and the metastasis must have histological features of benign ameloblastoma. This terminology follows that used for metastasizing pleomorphic adenoma which is included as a variant of conventional benign pleomorphic adenoma [39].

#### Odontogenic carcinomas

In the 2005 edition, an attempt was made to sub-classify ameloblastic carcinoma into three types: primary type, secondary type (dedifferentiated) intraosseous and secondary type (dedifferentiated) peripheral. This subclassification was felt to be unnecessarily complex for an already rare lesion and had no justification on behavioural grounds. In 2017, there is a single diagnostic entity of ameloblastic carcinoma, although the text recognises the varied histological features.

Similarly, primary intraosseous carcinoma (PIC) appears as a single diagnostic entity in 2017. In 2005, there was an attempt to divide it into three subtypes according to their putative origin from OKCs or from other odontogenic cysts. The new edition recognises that some PIC may arise from pre-existing cysts, but designation as specific subtypes was not necessary nor justified on clinicopathological grounds.

*Clear cell odontogenic carcinoma* remains as a distinct entity, but the text is updated to recognise recent studies showing that they harbour a EWSR1-ATF1 translocation [40, 41]. This translocation is seen in a number of clear cell lesions including salivary clear cell carcinoma. However, although not specific, when used in context, it is an important and useful molecular test which can be used to distinguish clear cell odontogenic carcinoma from other odontogenic lesions which may contain clear cells, including clear cell variant of calcifying epithelial odontogenic tumour and SOC.

#### The mixed odontogenic tumours

This group contains a number of lesions from true neoplasms (ameloblastic fibroma) to lesions that have been defined as hamartomas (odontomas). All are thought to be composed of both epithelial and mesenchymal elements and may show varied degrees of inductive change with formation of dental hard tissues. The new classification contains one new entity—the *primordial odontogenic tumour* described above. In addition, four lesions which were categorised as mixed tumours in 2005 have been deleted. These include calcifying cystic odontogenic tumour (which has been redesignated as COC) and odontoameloblastoma, which have been discussed previously. In addition, the ameloblastic fibrodentinoma and ameloblastic fibro-odontoma have been removed as distinct entities. The true nature of these lesions has long been debated, and it is now thought that they represent part of the spectrum of histological changes seen in a developing odontoma [42, 43].

In the new classification therefore, the odontomas and dentinogenic ghost cell tumours remain essentially the same, with updates on genetic and immunohistochemical findings. Similarly, the description of ameloblastic fibroma is essentially unchanged. The lesion is composed of a cellular mesenchymal component resembling the dental papilla, containing branching strands of bilayered columnar epithelium resembling dental lamina. Occasional buds are seen, with stellate reticulum like cells centrally, giving the appearance of the early developing enamel organ. These histological features are characteristic but not specific, since they may be seen in an early developing (non-calcifying) odontoma. In some cases, there may be evidence of inductive change, and dental hard tissue may be noted. Previously, if dentine was seen, the lesions were designated ameloblastic fibrodentinoma (AFD), and if dentine and enamel were seen, the lesion was termed ameloblastic fibro-odontoma (AFO). However, these features are indistinguishable from a developing odontoma, and it is considered that if lesions were left, they would continue to mature into fully calcified lesions [42, 43]. The consensus group therefore agreed to remove AFD and AFO from the classification, since they are “most likely developing odontomes” [44]. However, this still remains controversial, and it is recognised that some of these lesions may reach large sizes and arise in age groups which are not always consistent with a hamartoma. A recent review suggests that the clinicopathological features of these lesions do not always support the idea of progressive maturation into odontomas and that at least some AFOs and AFDs may be true neoplasms [45].

## Maxillofacial bone tumours

The new classification includes a number of bone tumours and related lesions (Table 3). Although the criteria for inclusion was not always clear, most are either important in the differential diagnosis of jaw lesions, have a propensity to arise within the jaw bones or have characteristic features when encountered at this site. All have been updated for this new edition, particularly with regard to the genetic profiles, but for the most part, the descriptions of these lesions remain similar to the 2005 edition or are the same as in the corresponding WHO classifications of tumours at other sites. The major changes with regard to maxillofacial pathology are clarification around the definition and terminology of ossifying fibromas and the cemento-osseous dysplasias.

**Table 3:** Bone tumours and related lesions

Malignant maxillofacial bone and cartilage tumours
Chondrosarcoma
Mesenchymal chondrosarcoma
Osteosarcoma
Benign maxillofacial bone and cartilage tumours
Chondroma
Osteoma
Melanotic neuroectodermal tumour of infancy
Chondroblastoma
Chondromyxoid fibroma
Osteoid osteoma
Osteoblastoma
Desmoplastic fibroma
Fibro- and chondro-osseous lesions
Ossifying fibroma
Familial gigantiform cementoma
Fibrous dysplasia
Cemento-osseous dysplasia
Osteochondroma
Giant cell lesions and bone cysts
Central giant cell granuloma
Peripheral giant cell granuloma
Cherubism
Aneurysmal bone cyst
Simple bone cyst
Haematolymphoid tumours
Solitary plasmacytoma

### Cemento-ossifying fibroma

Cemento-ossifying fibroma (COF) has been a confusing and ill-defined term for many years. In the 1st edition [1], the authors included two separate entities: *cementifying fibroma* as a type of “cementoma” and *ossifying fibroma* as a type of osteogenic neoplasm. The histological description of cementifying fibroma was what we now regard as a conventional ossifying fibroma [46, 47]. The 2nd edition [2], called both lesions *cemento-ossifying fibroma*, recognised the problem of a histological distinction of bone from cementum. In 2005 [3], the terminology changed again, and all the “cemento-ossifying fibromas” were regarded as *ossifying fibroma*. This was because cementum and bone are essentially the same tissue and can only be distinguished by their relationship to the tooth root [48]. When “cementum” is not attached to a tooth, it should no longer be considered as a specific tissue. However, there is a general consensus that when ossifying fibroma arises in the tooth-bearing areas, it is of odontogenic origin and arises within the periodontal ligament [46, 47]. In 2017, the consensus group felt that the term cemento-ossifying fibroma is suitably descriptive and indicates that these lesions are specific to the tooth-bearing areas of the jaws and can be distinguished from the two juvenile variants of ossifying fibroma [46, 49]. The new 4th edition therefore classifies cemento-ossifying fibroma as a benign mesenchymal odontogenic tumour (Table 2). This clearly distinguishes it from ossifying fibromas that are non-odontogenic and are classified under benign fibro- and chondro-osseous lesions (Table 3). The three variants are therefore defined as *cemento-ossifying fibroma*, *juvenile trabecular ossifying fibr*oma and *juvenile psammomatoid ossifying fibroma* [50].

### Cemento-osseous dysplasia

Cemento-osseous dysplasia (COD) has also been a controversial, much debated term. The arguments and discussions have been similar to those described above for ossifying fibromas. The 2005 edition defined these lesions as arising from periodontal tissues, but preferred to use the term “osseous dysplasia”, dropping “cemento” on the basis discussed above that cementum and bone are indistinguishable [48]. The new edition reverts back to the terminology *cemento-osseous dysplasia*, in order to recognise them as odontogenic with an origin in the periodontal ligament. Three variants are described: periapical COD, focal COD and florid COD [51]. Although many regard “gigantiform cementoma” as a variant of florid COD, the 2017 classification has retained *familial gigantiform cementum* as an entity. This is characterised by multiple, multiquadrant lesions which, in at least some cases, has a well-defined autosomal dominant inheritance pattern.

## Footnotes

1 The International consensus group met in Lyon in January 2016 and comprised: Prof Takashi Takata, Japan (Chair); Prof Daniel Baumhoer, Switzerland; Prof Samir El-Mofty, USA; Prof Edward Odell, UK; Prof Paul Speight, UK; Prof John Wright, USA, Prof Rosnah Zain, Malaysia
